# Emergence and dissemination of extensively drug-resistant ST307 Klebsiella pneumoniae in China

**DOI:** 10.1099/mgen.0.001718

**Published:** 2026-05-28

**Authors:** Weihua Han, Xinru Yuan, Can Yang, Shanshan Wang, Yin Zhou, Peiyao Zhou, Haojin Gao, Yu Huang, Zhixuan Chen, Jinjin Yang, Jiana Fu, Xinyi Fu, Jiawen Lou, Jiaqi Zou, Bingjie Wang, Fangyou Yu

**Affiliations:** 1 Department of Clinical Laboratory, Shanghai Pulmonary Hospital, School of Medicine, Tongji University, Shanghai, PR China; 2 Department of Clinical Laboratory, The First Affiliated Hospital of Ningbo University, Ningbo, Zhejiang, PR China

**Keywords:** carbapenem-resistant, colistin, *Klebsiella pneumoniae*, ST307, tigecycline

## Abstract

Carbapenem-resistant *Klebsiella pneumoniae* (CRKP) poses a significant threat to global public health. Identifying high-risk clones that facilitate the global dissemination of carbapenemases is essential for developing effective strategies to address this challenge. Here, we collected 29 ST307 CRKP isolates harbouring the *bla*_KPC-2_ and/or *bla*_NDM-5_ genes from three hospitals. *bla*_NDM-5_ was carried by the IncX3 plasmid, and *bla*_KPC-2_ was located on an IncFII plasmid. All of them were horizontally transmissible as verified by plasmid conjugation assays. Phylogenetic analysis revealed a clonal outbreak involving 27 of the isolates in this study and further demonstrated an emerging trend of *bla*_KPC-2_- and *bla*_NDM-5_-positive CRKP strains within the ST307 lineages in China since 2023. Resistance to ‘last-resort’ antibiotics mediated by genetic mutations is a major contributor to treatment failure in CRKP infections. In this study, we functionally confirmed that the plasmid-borne *tet*(A)^I235F^ mutation confers resistance to both tigecycline and eravacycline. The results of the murine bloodstream infection model further demonstrated that the *tet*(A)^I235F^ mutation increased the treatment cost of tigecycline. Furthermore, we found colistin resistance in ST307 CRKP primarily mediated by mutations in the *mgrB* and *pmrB* genes. Specific mutation patterns, including *mgrB* disruption by IS*5D*, *mgrB* W20* and *pmrB* S203P substitutions, were identified in nine colistin-resistant ST307 CRKP isolates in this study. These findings highlight the need for enhanced surveillance and control measures to prevent the potential widespread outbreak of extensively drug-resistant ST307 isolates in China.

## Data Summary

Genome sequences of the strains used in this study have been deposited in the GenBank database under BioProject number PRJNA1332962. The supporting data have been deposited in Figshare with the identifier https://doi.org/10.6084/m9.figshare.32048676.

**Impact Statement**

The worldwide distribution of extensively drug-resistant (XDR) *Klebsiella pneumoniae* is mainly due to the dissemination of high-risk clones carrying antimicrobial resistance genes and genetic mutations. Therefore, it is critically important to identify high-risk clones that facilitate the outbreak of XDR strains in clinical practice. Although outbreaks of ST307 *K. pneumoniae* carrying carbapenemases have been continuously reported in Western countries, a detailed mechanistic investigation of XDR ST307 *K. pneumoniae* isolated from China has been missing. Specifically, we collected 29 ST307 carbapenem-resistant *K. pneumoniae* (CRKP) isolates harbouring *bla*_KPC-2_ and/or *bla*_NDM-5_ in China and analysed their genetic features using whole-genome sequencing. The horizontal self-transfer of IncFII and IncX3 plasmids harbouring *bla*_KPC-2_ and *bla*_NDM-5_, respectively, contributes to the dissemination of ST307 CRKP. Genetically, these isolates were phylogenetically closely related, indicating that ST307 is a high-risk clone capable of causing nosocomial CRKP outbreaks. A plasmid carried the *tet*(A)^I235F^ mutation, which mediates resistance to tigecycline and eravacycline resistant, and the increased therapeutic cost was verified using a mouse infection model. Furthermore, mutations including *mgrB* disruption by IS*5D*, *mgrB* W20* and *pmrB* S203P were identified to cause colistin resistance in ST307 CRKP isolates. Our findings will provide substantial insights for clinical surveillance of XDR *K. pneumoniae* and nosocomial infection control.

## Introduction

*Klebsiella pneumoniae* is a critical pathogen causing severe complex infections, including pneumonia, liver abscess and bloodstream infections [1,2]. Most *K. pneumoniae* strains have been shown to acquire antibiotic resistance genes via mobile genetic elements (MGEs), particularly plasmid-borne carbapenemase genes [3]. The most prevalent carbapenemases are categorized into three groups within which five principal families can be distinguished according to Ambler classification: the Ambler class A group with *K. pneumoniae* carbapenemases (KPCs), the Ambler class B group with metallo-*β*-lactamases (VIM, IMP and NDM types) and the Ambler class D group with OXA-48-like oxacillinases, all of which have achieved global distribution [4]. The worldwide spread of carbapenemase-producing *K. pneumoniae* [carbapenem-resistant *K. pneumoniae* (CRKP)] has become a severe crisis in clinical settings for years and now constitutes a significant global public health problem [5]. Considerably, high-risk clones carrying antimicrobial resistance (AMR) genes (AMGs) have contributed to the worldwide distribution of CRKP. While *K. pneumoniae* sequence type (ST) 258 and ST11 dominate carbapenem resistance dissemination in the USA and China, respectively [6,7], new extensively drug-resistant (XDR) lineages have emerged in recent years [8,9]. Among them, *K. pneumoniae* ST307 harbouring carbapenemases to achieve multidrug-resistant (MDR) or XDR came into the spotlight.

Following its initial detection in the Netherlands in 2008, *K. pneumoniae* ST307 demonstrated rapid global spread, with subsequent outbreaks reported in Italy, Colombia, the USA (Texas) and South Africa [10]. Recent epidemiological studies indicate a concerning trend of worldwide ST307 expansion within carbapenemase-producing *K. pneumoniae* isolates, showing the potential to outcompete other high-risk AMR clones like ST258 in specific regions, such as Italy and Colombia [11,12]. Although *K. pneumoniae* ST307 is associated with various carbapenemases to achieve global dissemination, regional variations in predominant carbapenemase types remain evident. The ST307 *K. pneumoniae* strains carrying *bla*_KPC_, *bla*_OXA-181_, *bla*_NDM-1_ and *bla*_VIM-1_ caused outbreaks in the UK, South Africa, Mexico and Italy, respectively [13,16]. Unlike its established prevalence in these regions, ST307 CRKP is only sporadically reported in China. A previous study reported the occurrence of ST307 CRKP strains carrying both carbapenemase and extended-spectrum *β*-lactamase (ESBL) genes in Shenzhen [17]. Fourteen *bla*_IMP-38_-carrying ST307 *K. pneumoniae* strains were isolated from patients with neonatal sepsis [18]. NDM-5-producing ST307 *K. pneumoniae* strains caused an outbreak at a Shanghai teaching hospital [19]. In addition, ST307 *K. pneumoniae* strains carrying *mcr-1*, *bla*_OXA-48_ and *bla*_NDM-1_ genes have also been sporadically reported in China [20,21].

Tigecycline and its more effective derivative, eravacycline, exhibit potent antimicrobial activity against CRKP [22,23]. However, resistance to these antibiotics has been frequently reported since their introduction into clinical practice [24,25]. In terms of molecular resistance mechanisms, overexpression of resistance–nodulation–cell division-type efflux pumps, ribosomal protein mutations and plasmid-mediated *tet*(X) has been most commonly reported in *Enterobacterales* [26,28]. Several studies have identified tigecycline and eravacycline resistance in CRKP resulting from *tet*(A) mutation during tigecycline treatment [29,31], but functional validation is needed to verify their resistance-mediating effects. Colistin is another potent antibiotic against CRKP [32]. However, the use of colistin for the management of CRKP-associated infections has been accompanied by the emergence of colistin resistance [33]. The primary mechanism of colistin resistance involves lipopolysaccharide (LPS) modification mediated by intrinsic (chromosomally encoded) alterations in the two-component regulatory systems PhoPQ and PmrAB and their negative regulator MgrB [32]. Additionally, plasmid-mediated mechanisms via *mcr*-like genes are involved in LPS modification [34]. Both intrinsic and plasmid-mediated mechanisms induce the addition of cationic groups to LPS, specifically 4-amino-4-deoxy-l-arabinose (l-Ara4N) and/or phosphoethanolamine (PEtN), thereby diminishing the binding affinity of colistin to bacterial cell wall [32]. A previous study reported that colistin-resistant (Col-R) ST307 *K. pneumoniae* harbouring *bla*_OXA-48_-like determinants has become the dominant clinical clone in Moscow [35]. However, few studies have reported the prevalence of Col-R ST307 CRKP in China.

In this study, we characterized the drug-resistant and pathogenicity features of 29 ST307 CRKP strains isolated from 3 hospitals in coastal eastern China. All of these isolates were XDR isolates, carrying ESBL genes (*bla*_CTX-M-15_ and *bla*_SHV-28_), carbapenemase genes (*bla*_KPC-2_ and/or *bla*_NDM-5_) and other resistance genes associated with aminoglycoside, fluoroquinolone and *β*-lactam antibiotics. We described the phylogenetic relatedness among these 29 isolates and other globally isolated ST307 *K. pneumoniae* strains. Furthermore, we clarified the molecular mechanism underlying concurrent resistance to tigecycline and eravacycline in strain YF2075, as well as the colistin resistance mechanisms in Col-R ST307 CRKP isolates. Overall, our results underscore the urgency of implementing preemptive surveillance to mitigate the risk of a large-scale outbreak of this high-risk clone in China.

## Methods

### Bacterial strains, plasmids and culture conditions

Twenty-nine ST307 CRKP strains were isolated from three hospitals located in Shanghai or Ningbo. Detailed information is presented in Tables S1 and S2 (available in the online Supplementary Material). *Escherichia coli* DH5*α* competent cells used for plasmid transformation were brought from Sangon Biotech (B528413, Shanghai, China). All strains were cultured in Luria–Bertani (LB, Oxoid, Basingstoke, UK) medium at 37 °C with shaking at 220 r.p.m., or on LB agar plates at 37 °C. The pACYC184 plasmid and *E. coli* EC600 were gifts from Huashan Hospital (Shanghai, China) [36]. Comprehensive information on bacterial strains and plasmids used in this study is listed in Table S1.

### Antimicrobial susceptibility test

The minimum inhibitory concentrations (MICs) of meropenem, imipenem, ertapenem, cefoxitin, ceftazidime, ceftriaxone, cefuroxime, cefepime, cefotaxime, colistin, ceftazidime–avibactam, cefoperazone/sulbactam, piperacillin–tazobactam, amoxicillin/clavulanic acid, amikacin, levofloxacin and trimethoprim/sulfamethoxazole were determined by broth microdilution method according to the Clinical and Laboratory Standards Institute (CLSI) guidelines. Besides, the MICs of tigecycline (Autobio, Zhengzhou, China) and eravacycline (Liofilchem S.r.l., Italy) were determined by E-test assays. *E. coli* ATCC25922 was used as quality control for the antimicrobial susceptibility test (AST). The MICs were interpreted according to CLSI breakpoints [37].

### Whole-genome sequencing and bioinformatic analysis

Genomic DNA was extracted using a commercial kit (Qiagen, Hilden, Germany), according to the manufacturer’s protocols. Isolates were sequenced using the Illumina NovaSeq 6000 platform, and a representative strain (*K. pneumoniae* strain YF2075) was further sequenced using the long-read PacBio Sequel platform. The assembled genome sequences were annotated by Prokka v1.14.6 [38]. The acquired antibiotic resistance genes, virulence determinants, serotype predictions, multilocus sequence type (MLST) and plasmid replicon types were analysed using ResFinder v4.7.2 [39,40, 40], Kleborate v3.2.4 [41], MLST v2.23.0[42] and PlasmidFinder v3.0.2 [43] databases, respectively. Alignments of plasmids were generated by Proksee [44], while alignments of genetic structures were performed by Easyfig_2.2.5 _win software [45]. To explore the global distribution of *K. pneumoniae* ST307, all genome sequences of *K. pneumoniae* (*n*=13,308) were downloaded from the NCBI Pathogen Detection database (data cutoff was July 2025) and further analysed by MLST v2.23.0. Subsequently, the data of a total of 1,701 ST307 isolates supplemented with the 29 genomes sequenced in this study (deposited in the NCBI BioProject database under accession no. PRJNA1332962) were used to construct the core phylogenetic tree by FastTree v2.1.11 [46] with the approximately maximum-likelihood method. The genome YF2075 was used as the reference template, and all genomes were mapped to the reference genome. Furthermore, core genome SNP typing was performed with Snippy v4.6.0 (github.com/tseemann/snippy), and the tree was visualized and annotated via iTOL v7.2.1.

### Conjugation assay

The donors and recipients were cultured in LB broth to the logarithmic phase at 37 °C. Then, they were mixed in a 1:1 ratio, centrifuged at 8,000 ***g*** for 2 min and resuspended in 20 µl 10 mM MgSO_4_. The suspension was spotted on an LB plate and incubated at 37 °C overnight. Subsequently, the serial dilutions were plated in an LB plate containing appropriate antibiotics [meropenem, 2 µg ml^−1^ (*bla*_KPC-2_, *bla*_NDM-5_); rifampicin, 600 µg ml^−1^ (*E. coli* EC600 recipient)]. Bacterial identification was carried out using a VITEK MS PRIME system with Myla Knowledge Base v3.2 software (bioMérieux, Marcy l’Etoile, France). The number of transconjugants per donor was calculated to determine the conjugation frequency. Besides, PCR was conducted to confirm the presence of *bla*_KPC-2_ and *bla*_NDM-5_ genes (Fig. S1). Corresponding primers and PCR amplification conditions were described in Table S3.

### Plasmid construction and transformation

Purified pACYC184 plasmid DNA was extracted from liquid broth cultures by using the TIANprep Mini Plasmid Kit (TIANGEN, Beijing, China). The *tet*(A) and *tet*(A)^I235F^ genes, which contained their original promoter sequences, were amplified from the genomes of *K. pneumoniae* YF2190 and YF2075, respectively. The primers used in this assay are listed in Table S3. The PCR products were recombined into the pACYC184 vector using NEBuilder HiFi DNA Assembly Master Mix (New England Biolabs, Ipswich, MA, USA), which was further transformed into *E. coli* strain DH5*α* via chemical transformation experiments (Fig. S2). Positive transformants were selected on LB agar plates containing 10 µg ml^−1^ chloramphenicol and verified by *tet*(A) and *tet*(A)^I235F^ genes PCR and Sanger sequencing. The constructed plasmids were extracted from DH5α and subsequently electroporated into *K. pneumoniae* YF2164.

### Growth curve

Overnight cultures of *K. pneumoniae* were adjusted to a turbidity equivalent to that of the McFarland standard 0.5, diluted 1:200 in fresh LB medium and incubated at 37 °C with shaking (220 r.p.m.) for 24 h. The OD at 600 nm (OD_600_) was measured every 30 min for 24 h using a microplate reader (Oy Growth Curves Ab Ltd, Helsinki, Finland). All experiments were performed in triplicate.

### Murine bloodstream infection model

Six-week-old female BALB/c mice were randomly assigned into four groups, each containing six mice. The mice were housed in a specific pathogen-free facility and fed an *ad libitum* diet. Overnight cultures of *K. pneumoniae* YF2164-pACYC184 and YF2164-pACYC184-*tet*(A)^I235F^ were diluted 1:100 in fresh LB medium and incubated at 37 °C with shaking (220 r.p.m.) for 4 h to the logarithmic growth phase. After harvested by centrifugation at 4,000 ***g*** for 5 min, the bacteria were washed twice and adjusted to an OD_600_ equal to 1.0. Then, 100 µl *K. pneumoniae* bacterial suspension (1×10^9^ c.f.u. ml^−1^) was injected into mice via the tail vein. After 2 h post-infection, 5 mg kg^−1^ tigecycline was administered to the treatment group, and DMSO was used as control. Mice were euthanized at 24 h, and visceral organs including heart, liver, spleen, lung and kidney were aseptically excised and homogenized in sterile PBS. The bacterial loads in the specific organs were determined by serial dilutions and spotted on LB plates.

### Total RNA extraction, cDNA synthesis and real-time PCR

Bacteria were grown to mid-exponential phase at 37 °C in LB medium. After centrifugation at 12,000 ***g*** for 2 min, cells were resuspended in Tris-EDTA (TE) buffer containing 20 mg ml^−1^ lysozyme and incubated at 37 °C for 20 min. After digestion, total RNA was extracted according to the manufacturer’s instructions (Spin Column Bacteria Total RNA Purification Kit; Sangon Biotech, Shanghai, China). RNA was reverse transcribed into cDNA using a cDNA synthesis kit (Takara, Tokyo, Japan). Quantitative real-time PCR was performed using TB Green Master Mix (Takara). The primers used for RT-qPCR are listed in Table S3. Relative expression values were calculated using the 2^-∆∆CT^ method with the stable housekeeping gene *rpoD* as the normalizer.

### ***Galleria mellonella*** infection model

We used a *G. mellonella* killing assay to evaluate the pathogenicity of *K. pneumoniae* ST307 CRKP. *G. mellonella* caterpillars were stored at 4 °C before use; after regaining activity at 37 °C for 2 h, each group of the caterpillars was inoculated with 10 µl of at a concentration of 1.0×10^7^ c.f.u. ml^−1^ *K*. *pneumoniae* ST307, NUTH-K2044 and HS11286. The saline-treated group was used as the control. The caterpillars were placed in Petri dishes at 37 °C, and the survival rates were recorded every 6 h.

### Quantitative siderophore production assay

To assess the ability of bacterial supernatants to chelate iron, a quantitative siderophore production assay was applied following established protocols [47]. Briefly, 1 µl of logarithmic-phase, iron-chelated cultures was spotted on chrome azurol S (CAS) plates. After incubation at 37 °C for 24 h, the diameters of orange halos were measured to identify siderophore production.

### Serum resistance assay

The serum resistance assay was performed to assess the ability of ST307 CRKP strains to resist the killing of serum. The mid-log phase bacterial cells (1.5×10^7^ c.f.u. ml^−1^) were mixed with human serum collected from healthy volunteers at a 1:3 ratio and incubated at 37 °C for 2 h. After serial dilution, bacteria were plated on LB agar and cultured at 37 °C for 12 h for the enumeration of viable bacteria. Informed consent from the donors was obtained for the use of serum.

### Statistical analysis

GraphPad Prism v8.00 software was used to conduct statistical analysis. Two-tailed Student’s *t*-test was used to analyse the statistical difference between two groups, and a one-way ANOVA was used to analyse the statistical difference between multiple groups after passing the normal distribution test. Error bars in the figures represent the sd of the dataset (mean±sd). *P<0.05, **P<0.01, ***P<0.001 and ****P<0.0001.

## Results

### General clinical characteristics of ST307 CRKP

**Fig. 1.**
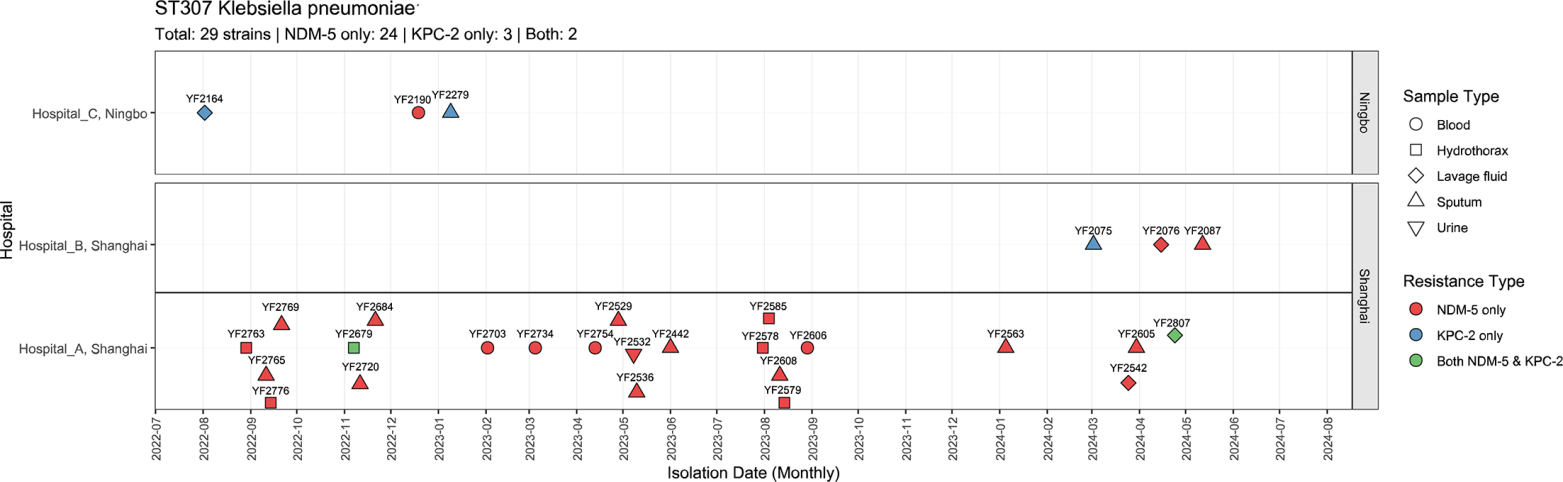
Timeline scatter plot of 29 ST307 *K. pneumoniae* strains across three hospitals in Ningbo and Shanghai from mid-2022 to mid-2024. Sample types are represented by different shapes, and resistance types are shown in different colors.

As shown in Fig. 1, a total of 29 ST307 CRKP isolates were collected from August 2022 to June 2024 at 3 hospitals located in Shanghai and Ningbo. The 29 isolates were derived from a wide range of clinical sources, including sputum (*n*=13), lavage fluid (*n*=4), hydrothorax (*n*=6), blood (*n*=5) and urine (*n*=1) (Table S2). Notably, after the first ST307 CRKP strain (YF2763) was isolated from the hydrothorax of a patient hospitalized in the thoracic surgery ward, three additional ST307 CRKP strains were isolated from patients hospitalized in this ward between August 2022 and September 2023. During this period, the majority of ST307 CRKP isolates (73.68%, 14/19) from hospital A were obtained from the post-operative monitoring ward, suggesting the possibility of an outbreak between these two wards (Table S2). Subsequently, we detected four ST307 CRKP isolates (YF2563, YF2542, YF2605 and YF2807) from a patient who underwent lung transplantation and was hospitalized in the thoracic surgery ward at hospital A for 6 months (Fig. 1, Table S2). At hospital B, the first ST307 CRKP (YF2075) was identified from a patient in the intensive care unit, and the additional two ST307 CRKP strains were collected from patients on the same ward over the following 2 months (Fig. 1, Table S2). Three additional ST307 CRKP isolates collected from hospital C emerged between August 2022 and January 2023 (Fig. 1).

A panel of 29 isolates was obtained from 26 patients with severe pulmonary disease, including interstitial lung disease (*n*=8), respiratory failure (*n*=2), lung transplantation status (*n*=5), chronic obstructive pulmonary disease (*n*=2), pulmonary arterial hypertension (*n*=1), pneumonia (*n*=3) and other diseases such as infectious fever (*n*=1), septic shock (*n*=1), sepsis (*n*=1), cancer (*n*=1) and pulmonary ground-glass opacity (*n*=1) (Table S2). The median age of the 26 patients (18 males and 8 females) was 66 years (range, 20–89). Importantly, except for one outpatient patient, all patients had received carbapenem antimicrobials (meropenem and/or imipenem) before CRKP detection. The patient who tested positive for YF2075 had previously received tigecycline treatment.

### Antimicrobial susceptibility and resistance gene profiles

We performed ASTs to clarify the antibacterial resistance profiles of these 29 isolates. Table 1 illustrates that these 29 ST307 *K. pneumoniae* were typical multidrug resistant strains with resistance to meropenem (MIC, ≥ 64 µg ml^−1^), imipenem (MIC, ≥ 16 µg ml^−1^), ertapenem (MIC, ≥ 8 µg ml^−1^), cefoxitin (MIC, ≥ 64 µg ml^−1^), ceftazidime (MIC, ≥ 32 µg ml^−1^), ceftriaxone (MIC, ≥ 64 µg ml^−1^), cefuroxime (MIC, ≥ 64 µg ml^−1^), cefepime (MIC, ≥ 32 µg ml^−1^), cefoperazone/sulbactam (MIC, ≥ 64 µg ml^−1^), piperacillin–tazobactam (MIC, ≥ 128 µg ml^−1^), amoxicillin/clavulanic acid (MIC, ≥ 32 µg ml^−1^), levofloxacin (MIC, ≥ 4 µg ml^−1^) and trimethoprim/sulfamethoxazole (MIC, ≥ 320 µg ml^−1^). In addition, two isolates were susceptible to ceftazidime–avibactam (MIC, 2 µg ml^−1^), and 26 isolates were resistant (MIC, ≥512/4 µg ml^−1^) (Table 1). Besides, the isolate named YF2279 is intermediately resistant to ceftazidime–avibactam (MIC, 16 µg ml^−1^) (Table 1). Moreover, 24.14% (7/29) isolates showed resistance to amikacin (MIC, ≥ 64 µg ml^−1^), and 34.48% (10/29) isolates showed resistance to colistin (MIC, 4–32 µg ml^−1^) (Table 1). Apart from the YF2075 strain (an isolate resistant to tigecycline and eravacycline with an MIC of 12 µg ml^−1^ and 6 µg ml^−1^, respectively), the other 28 strains were all susceptible to tigecycline and eravacycline (Table 1).

**Table 1.** Antimicrobial drug susceptibility profiles of ST307 CRKP in this study

Isolate	MIC (μg ml^−1^)																	
	**MEM**	**IPM**	**ETP**	**FOX**	**CAZ**	**CRO**	**CXM**	**FEP**	**CZA**	**CSL**	**TZP**	**AMC**	**AMK**	**LVX**	**TGC**	**ERV**	**COL**	**SXT**
**YF2075**	≥16 (R)	≥16 (R)	≥8 (R)	≥64 (R)	32 (R)	≥64 (R)	≥64 (R)	≥32 (R)	2/4 (S)	≥64 (R)	≥128 (R)	≥32 (R)	4 (S)	≥8 (R)	12 (R)	6 (R)	0.25 (S)	≥320 (R)
**YF2076**	≥16 (R)	≥16 (R)	≥8 (R)	≥64 (R)	≥64 (R)	≥64 (R)	≥64 (R)	≥32 (R)	≥512/4 (R)	≥64 (R)	≥128 (R)	≥32 (R)	≤2 (S)	≥8 (R)	0.5 (S)	0.5 (S)	0.25 (S)	≥320 (R)
**YF2087**	≥16 (R)	≥16 (R)	≥8 (R)	≥64 (R)	≥64 (R)	≥64 (R)	≥64 (R)	≥32 (R)	≥512/4 (R)	≥64 (R)	≥128 (R)	≥32 (R)	≤2 (S)	≥8 (R)	0.5 (S)	0.5 (S)	0.25 (S)	≥320 (R)
**YF2164**	≥16 (R)	≥16 (R)	≥8 (R)	≥64 (R)	≥64 (R)	≥64 (R)	≥64 (R)	≥32(R)	2/4 (S)	≥64 (R)	≥128 (R)	≥32 (R)	≤2 (S)	≥8(R)	0.25 (S)	0.19 (S)	0.25 (S)	≥320 (R)
**YF2190**	≥16 (R)	≥16 (R)	≥8 (R)	≥64 (R)	≥64 (R)	≥64 (R)	≥64 (R)	≥32(R)	≥512/4 (R)	≥64 (R)	≥128 (R)	≥32 (R)	4 (S)	≥8 (R)	0.5 (S)	0.5 (R)	0.25(S)	≥320 (R)
**YF2279**	≥16 (R)	≥16 (R)	≥8 (R)	≥64 (R)	≥64 (R)	≥64 (R)	≥64 (R)	≥32 (R)	16/4 (I)	≥64 (R)	≥128 (R)	≥32 (R)	≤2 (S)	4 (R)	0.5 (S)	0.5 (S)	0.25(S)	≥320 (R)
**YF2442**	≥16 (R)	≥16 (R)	≥8 (R)	≥64 (R)	≥64 (R)	≥64 (R)	≥64 (R)	≥32 (R)	≥512/4 (R)	≥64 (R)	≥128 (R)	≥32 (R)	≥64 (R)	≥8 (R)	0.5 (S)	0.5 (S)	64(R)	≥320 (R)
**YF2529**	≥16 (R)	≥16 (R)	≥8 (R)	≥64 (R)	≥64 (R)	≥64(R)	≥64(R)	≥32 (R)	≥512/4 (R)	≥64 (R)	≥128 (R)	≥32 (R)	≤2 (S)	≥8 (R)	0.5 (S)	0.5 (S)	0.25 (S)	≥320 (R)
**YF2532**	≥16 (R)	≥16 (R)	≥8 (R)	≥64 (R)	≥64 (R)	≥64 (R)	≥64 (R)	≥32 (R)	≥512/4 (R)	≥64 (R)	≥128 (R)	≥32 (R)	≤2 (S)	≥8 (R)	0.5 (S)	0.5 (S)	0.25 (S)	≥320 (R)
**YF2536**	≥16 (R)	≥16 (R)	≥8 (R)	≥64 (R)	≥64 (R)	≥64 (R)	≥64 (R)	≥32 (R)	≥512/4 (R)	≥64 (R)	≥128 (R)	≥32 (R)	≤2 (S)	≥8 (R)	0.5 (S)	0.5 (S)	0.25 (S)	≥320 (R)
**YF2542**	≥16 (R)	≥16 (R)	≥8 (R)	≥64 (R)	≥64 (R)	≥64 (R)	≥64 (R)	≥32 (R)	≥512/4 (R)	≥64 (R)	≥128 (R)	≥32 (R)	≤2 (S)	≥8 (R)	0.5 (S)	0.5 (S)	32 (R)	≥320 (R)
**YF2563**	≥16 (R)	≥16 (R)	≥8 (R)	≥64 (R)	≥64 (R)	≥64 (R)	≥64 (R)	≥32 (R)	≥512/4 (R)	≥64 (R)	≥128 (R)	≥32 (R)	≥64 (R)	≥8(R)	0.5 (S)	0.5 (S)	0.25 (S)	≥320 (R)
**YF2578**	≥16 (R)	≥16 (R)	≥8 (R)	≥64 (R)	≥64 (R)	≥64 (R)	≥64 (R)	≥32 (R)	≥512/4 (R)	≥64 (R)	≥128 (R)	≥32 (R)	≤2 (S)	≥8 (R)	0.5 (S)	0.5 (S)	8 (R)	≥320 (R)
**YF2579**	≥16 (R)	≥16 (R)	≥8 (R)	≥64 (R)	≥64 (R)	≥64 (R)	≥64 (R)	≥32 (R)	≥512/4 (R)	≥64 (R)	≥128 (R)	≥32 (R)	≤2 (S)	≥8 (R)	0.5(S)	0.5 (S)	8 (R)	≥320 (R)
**YF2585**	≥16 (R)	≥16 (R)	≥8 (R)	≥64 (R)	≥64 (R)	≥64 (R)	≥64 (R)	≥32 (R)	≥512/4 (R)	≥64 (R)	≥128 (R)	≥32 (R)	≤2 (S)	≥8 (R)	0.5 (S)	0.5 (S)	16 (R)	≥320 (R)
**YF2605**	≥16 (R)	≥16 (R)	≥8 (R)	≥64 (R)	≥64 (R)	≥64 (R)	≥64 (R)	≥32 (R)	≥512/4 (R)	≥64 (R)	≥128 (R)	≥32 (R)	≥64 (R)	≥8 (R)	0.5 (S)	0.5 (S)	0.25 (S)	≥320 (R)
**YF2606**	≥16 (R)	≥16 (R)	≥8 (R)	≥64 (R)	≥64 (R)	≥64 (R)	≥64 (R)	≥32 (R)	≥512/4 (R)	≥64 (R)	≥128 (R)	≥32 (R)	≤2 (S)	≥8 (R)	0.5 (S)	0.5 (S)	8 (R)	≥320 (R)
**YF2608**	≥16 (R)	≥16 (R)	≥8 (R)	≥64 (R)	≥64 (R)	≥64 (R)	≥64 (R)	≥32 (R)	≥512/4 (R)	≥64 (R)	≥128 (R)	≥32 (R)	≤2 (S)	≥8 (R)	0.5 (S)	0.5 (S)	8 (R)	≥320 (R)
**YF2679**	≥16 (R)	≥16 (R)	≥8 (R)	≥64 (R)	≥64 (R)	≥64 (R)	≥64 (R)	≥32 (R)	≥512/4 (R)	≥64 (R)	≥128 (R)	≥32 (R)	≥64 (R)	≥8 (R)	0.5 (S)	0.5 (S)	0.25 (S)	≥320 (R)
**YF2684**	≥16 (R)	≥16 (R)	≥8 (R)	≥64 (R)	≥64 (R)	≥64 (R)	≥64 (R)	≥32 (R)	≥512/4 (R)	≥64 (R)	≥128 (R)	≥32 (R)	4 (S)	≥8 (R)	0.5 (S)	0.5 (S)	0.25 (S)	≥320 (R)
**YF2703**	≥16 (R)	≥16 (R)	≥8 (R)	≥64 (R)	≥64 (R)	≥64 (R)	≥64 (R)	≥32 (R)	≥512/4 (R)	≥64 (R)	≥128 (R)	≥32 (R)	≤2 (S)	≥8 (R)	0.5 (S)	0.5 (S)	0.25 (S)	≥320 (R)
**YF2720**	≥16 (R)	≥16 (R)	≥8 (R)	≥64 (R)	≥64 (R)	≥64 (R)	≥64 (R)	≥32 (R)	≥512/4 (R)	≥64 (R)	≥128 (R)	≥32 (R)	≤2 (S)	≥8 (R)	0.5 (S)	0.5 (S)	32 (R)	≥320 (R)
**YF2734**	≥16 (R)	≥16 (R)	≥8 (R)	≥64 (R)	≥64 (R)	≥64 (R)	≥64 (R)	≥32 (R)	≥512/4 (R)	≥64 (R)	≥128 (R)	≥32 (R)	≤2 (S)	≥8 (R)	0.5 (S)	0.5 (S)	2 (I)	≥320 (R)
**YF2754**	≥16 (R)	≥16 (R)	≥8 (R)	≥64 (R)	≥64 (R)	≥64 (R)	≥64 (R)	≥32 (R)	≥512/4 (R)	≥64 (R)	≥128 (R)	≥32 (R)	≤2 (S)	≥8 (R)	0.5 (S)	0.5 (S)	1 (S)	≥320 (R)
**YF2763**	≥16 (R)	≥16 (R)	≥8 (R)	≥64 (R)	≥64 (R)	≥64 (R)	≥64 (R)	≥32 (R)	≥512/4 (R)	≥64 (R)	≥128 (R)	≥32 (R)	≥64 (R)	≥8 (R)	0.5 (S)	0.5 (S)	2 (I)	≥320 (R)
**YF2765**	≥16 (R)	≥16 (R)	≥8 (R)	≥64 (R)	≥64 (R)	≥64 (R)	≥64 (R)	≥32 (R)	≥512/4 (R)	≥64 (R)	≥128 (R)	≥32 (R)	4 (S)	≥8 (R)	0.5 (S)	0.5 (S)	2 (I)	≥320 (R)
**YF2769**	≥16 (R)	≥16 (R)	≥8 (R)	≥64 (R)	≥64 (R)	≥64 (R)	≥64 (R)	≥32 (R)	≥512/4 (R)	≥64 (R)	≥128 (R)	≥32 (R)	8 (S)	≥8 (R)	0.5 (S)	0.5 (S)	2 (I)	≥320 (R)
**YF2776**	≥16 (R)	≥16 (R)	≥8 (R)	≥64 (R)	≥64 (R)	≥64 (R)	≥64 (R)	≥32 (R)	≥512/4 (R)	≥64 (R)	≥128 (R)	≥32 (R)	≥64 (R)	≥8 (R)	0.5 (S)	0.5 (S)	4 (R)	≥320 (R)
**YF2807**	≥16 (R)	≥16 (R)	≥8 (R)	≥64 (R)	≥64 (R)	≥64 (R)	≥64 (R)	≥32 (R)	≥512/4 (R)	≥64 (R)	≥128 (R)	≥32 (R)	≥64 (R)	≥8 (R)	0.5 (S)	0.5 (S)	16 (R)	≥320 (R)
**ATCC25922**	≤0.25 (S)	≤0.25 (S)	≤0.12 (S)	≤4 (S)	0.5 (S)	≤0.25 (S)	4 (S)	≤0.12 (S)	0.5/4 (S)	≤8/128 (S)	≤4/4 (S)	4 (S)	4 (S)	0.015 (S)	2 (S)	0.032 (S)	0.25 (S)	≤20 (S)

AMC, amoxicillin/clavulanic acid; AMK, amikacin; CAZ, ceftazidime; COL, Colistin; CRO, ceftriaxone; CSL, cefoperazone/sulbactam; CXM, cefuroxime; CZA, ceftazidime–avibactam; ERV, eravacycline; ETP, ertapenem; FEP, cefepime; FOX, cefoxitin; IPM, imipenem; LVX, levofloxacin; MEM, meropenem; SXT, trimethoprim/sulfamethoxazole; TGC, tigecycline; TZP, piperacillin–tazobactam.

**Fig. 2.**
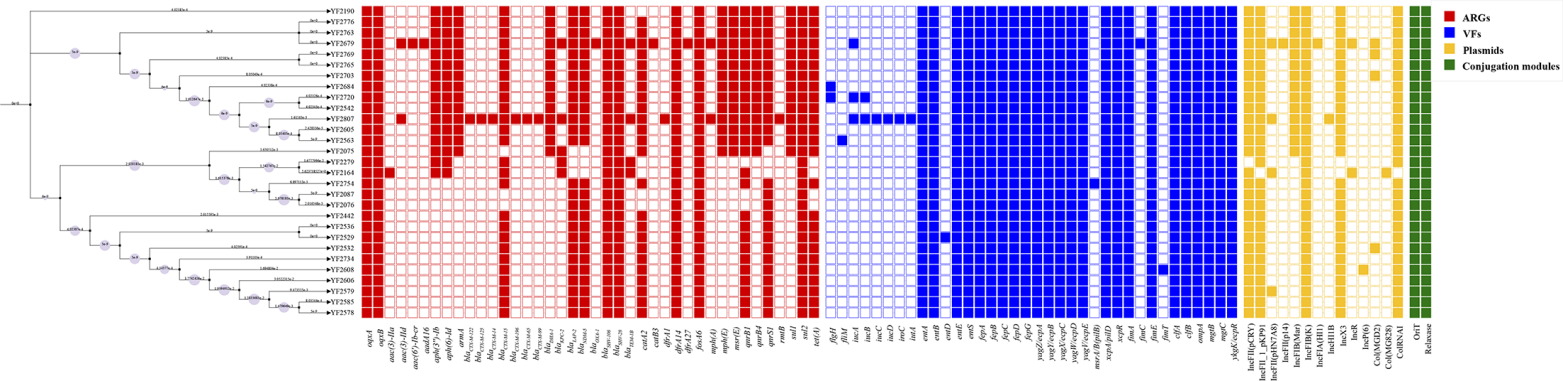
Heatmap with phylogenetic tree of ST307 CRKP isolates showing presence or absence of ARGs, virulence factors (VFs), plasmid types and conjugation modules across strains. The solid circles on branches represent bootstrap values. Larger circles indicate higher statistical support for the corresponding node.

Subsequently, the resistance gene profiles were further characterized using whole-genome sequencing (WGS) data. As shown in Fig. 2, 17.24% (5/29) isolates carried *bla*_KPC-2_, and 89.66% (26/29) strains produced NDM-5. Notably, we observed the coexistence of *bla*_KPC-2_ and *bla*_NDM-5_ in YF2679 and YF2807, which confer resistance to both carbapenems and ceftazidime/avibactam. These 29 strains all carried *oqxA*, *oqxB*, *bla*_SHV-28_, *bla*_SHV-106_, *dfrA14*, *fosA6* and *sul2*, and other resistance genes associated with resistance to *β*-lactams (*bla*_CTX-M-122_, *bla*_CTX-M-125_, *bla*_CTX-M-14_, *bla*_CTX-M-15_, *bla*_CTX-M-196_, *bla*_CTX-M-65_, *bla*_CTX-M-99_, *bla*_DHA-1_, *bla*_LAP-2_, *bla*_OXA-1_, *bla*_SHV-106_, *bla*_SHV-28_ and *bla*_TEM-1B_), aminoglycosides [*aac(3)-IIa*, *aac(3)-IId*, *aph(6″)-Ib-cr*, *aadA16*, *aph(3″)-Ib*, *aph(6)-Id*, *armA and rmtB*], quinolones (*qnrB1*, *qnrB4* and *qnrS1*), macrolides [*msr(E*), *mph(A*) and *mph(E*)], trimethoprim (*dfrA1* and *dfrA27*), tetracycline [*tet*(A)] and sulfonamides (*sul1*) were also detected in different isolates.

### The *bla*_KPC-2_ and *bla*_NDM-5_ carrying plasmids from ST307 CRKP can be successfully transferred into ***E. coli*** EC600

Plasmid conjugation is an essential mechanism driving the dissemination of antibiotic resistance in *Enterobacterales*. To determine whether ST307 CRKP acquired *bla*_KPC-2_- and *bla*_NDM-5_-carrying plasmids via plasmid-mediated horizontal transfer, we first used oriTfinder (oriTDB) [48] to predict the presence of complete conjugation modules in these strains. All isolates carried the DNA at the origin of transfer (*oriT*) and the relaxase gene, indicating the feasibility of plasmid transfer (Fig. 2). Therefore, conjugation assays were subsequently performed to evaluate plasmid transferability. The assays demonstrated successful horizontal transfer of *bla*_KPC-2_- or *bla*_NDM-5_-carrying plasmids to recipient strain *E. coli* EC600, with transfer frequencies 8.7×10^−3^ (YF2075), 2.2×10^−5^ (YF2076), 1.1×10^−3^ (YF2087), 9×10^−5^ (YF2164), 4.3×10^−4^ (YF2190) and 1.1×10^−4^ (YF2279), respectively. Remarkably, we obtained transconjugants containing *bla*_KPC-2_ only or both *bla*_KPC-2_ and *bla*_NDM-5_ from YF2807, with transfer frequencies 5.2×10^−4^ (*bla*_KPC-2_) and 2.8×10^−4^ (*bla*_NDM-5_), suggesting that *bla*_KPC-2_ and *bla*_NDM-5_ were carried by YF2807 via different plasmids. Furthermore, AST confirmed the acquisition of carbapenem resistance phenotypes in all transconjugants (Table 2). All transconjugants exhibited resistance to carbapenems (meropenem, imipenem and ertapenem), cephalosporins (cefoxitin, ceftazidime, ceftriaxone, cefuroxime and cefepime) and *β*-lactam/*β*-lactamase inhibitor combinations (cefoperazone/sulbactam, piperacillin–tazobactam and amoxicillin/clavulanic acid) (Table 2). Notably, the transconjugants carrying *bla*_NDM-5_ exhibited resistance to ceftazidime–avibactam, while the transconjugants only carrying *bla*_KPC-2_ were sensitive to ceftazidime–avibactam (Table 2). The plasmid carrying *bla*_NDM-5_ from YF2087 was subsequently extracted and sequenced by the Illumina NovaSeq 6000 platform (Sangon Biotech). The complete sequence of plasmid pYF2087 has been deposited in the GenBank database under accession number PZ277058. Analysis using Plasmidfinder v3.0.2 indicated that *bla*_NDM-5_ was located on an IncX3 plasmid (Fig. S3), which is a commonly reported plasmid type harbouring *bla*_NDM_ in China [49]. Taken together, these results indicate that ST307 CRKP harbours plasmids carrying carbapenemase genes acquired via horizontal conjugative transfer.

**Table 2.** Antimicrobial drug susceptibility profiles of *E. coli* EC600 transconjugants

Antibiotic	MIC (μg ml^−1^)								
	***E. coli* EC600 (*bla*_KPC-2_ from YF2075**)	***E. coli* EC600 (*bla*_NDM-5_ from YF2076**)	***E. coli* EC600 (*bla*_NDM-5_ from YF2087**)	***E. coli* EC600 (*bla*_KPC-2_ from YF2164**)	***E. coli* EC600 (*bla*_NDM-5_ from YF2190**)	***E. coli* EC600 (*bla*_KPC-2_ from YF2279**)	***E. coli* EC600 (*bla*_KPC-2_ and *bla_NDM-5_* from YF2807**)	***E. coli* EC600 (*bla*_KPC-2_ from YF2807**)	***E. coli* EC600**
**MEM**	4 (R)	8 (R)	8 (R)	64 (R)	16 (R)	16 (R)	16 (R)	16 (R)	0.25 (S)
**IPM**	≥16 (R)	≥16 (R)	≥16 (R)	≥16 (R)	≥16 (R)	≥16 (R)	≥16 (R)	≥16 (R)	≤0.25 (S)
**ETP**	≥8 (R)	≥8 (R)	≥8 (R)	≥8 (R)	≥8 (R)	≥8 (R)	≥8 (R)	≥8 (R)	≤0.12 (S)
**FOX**	32 (R)	≥64 (R)	≥64 (R)	≥64 (R)	≥64 (R)	≥64 (R)	≥64 (R)	≥64 (R)	8 (S)
**CAZ**	32 (R)	≥64 (R)	≥64 (R)	32 (R)	≥64 (R)	≥64 (R)	≥64 (R)	≥64 (R)	0.5 (S)
**CRO**	≥64 (R)	≥64 (R)	≥64 (R)	≥64 (R)	≥64 (R)	≥64 (R)	≥64 (R)	≥64 (R)	≤0.25 (S)
**CXM**	≥64 (R)	≥64 (R)	≥64 (R)	≥64 (R)	≥64 (R)	≥64 (R)	≥64 (R)	≥64 (R)	16 (I)
**FEP**	≥32(R)	16 (R)	16 (R)	≥32 (R)	16 (R)	16 (R)	16 (R)	16 (R)	≤0.12 (S)
**CZA**	0.5/4 (S)	≥256/4 (R)	≥256/4 (R)	0.5/4 (S)	≥256/4 (R)	1/4 (S)	≥256/4 (R)	0.5/4 (S)	0.25/4 (S)
**CSL**	≥64 (R)	≥64 (R)	≥64 (R)	≥64 (R)	≥64 (R)	≥64 (R)	≥64 (R)	≥64 (R)	≤8 (S)
**TZP**	≥128 (R)	≥128 (R)	≥128 (R)	≥128 (R)	≥128 (R)	≥128 (R)	≥128 (R)	≥128 (R)	≤4 (S)
**AMC**	≥32 (R)	≥32 (R)	≥32 (R)	≥32 (R)	≥32 (R)	≥32 (R)	≥32 (R)	≥32 (R)	4 (S)
**AMK**	≤2 (S)	≤2 (S)	≤2 (S)	4 (S)	≤2 (S)	≤2 (S)	≤2 (S)	≤2 (S)	≤2 (S)
**LVX**	0.5 (S)	0.5 (S)	0.5 (S)	0.5 (S)	0.5 (S)	0.5 (S)	0.5 (S)	0.5 (S)	0.5 (S)
**TGC**	≤0.5 (S)	≤0.5 (S)	≤0.5 (S)	≤0.5 (S)	≤0.5 (S)	≤0.5 (S)	≤0.5 (S)	≤0.5 (S)	≤0.5(S)
**SXT**	≤20 (S)	≤20 (S)	≤20 (S)	≤20 (S)	≤20 (S)	≤20 (S)	≤20 (S)	≤20 (S)	≤20(S)

AMC, amoxicillin/clavulanic acid; AMK, amikacin; CAZ, ceftazidime; CRO, ceftriaxone; CSL, cefoperazone/sulbactam; CXM, cefuroxime; CZA, ceftazidime–avibactam; ETP, ertapenem; FEP, cefepime; FOX, cefoxitin; IPM, imipenem; LVX, levofloxacin; MEM, meropenem; SXT, trimethoprim/sulfamethoxazole; TGC, tigecycline; TZP, piperacillin–tazobactam.

### The Tet(A)^I235F^ mutation confers tigecycline and eravacycline resistance in strain YF2075

**Fig. 3.**
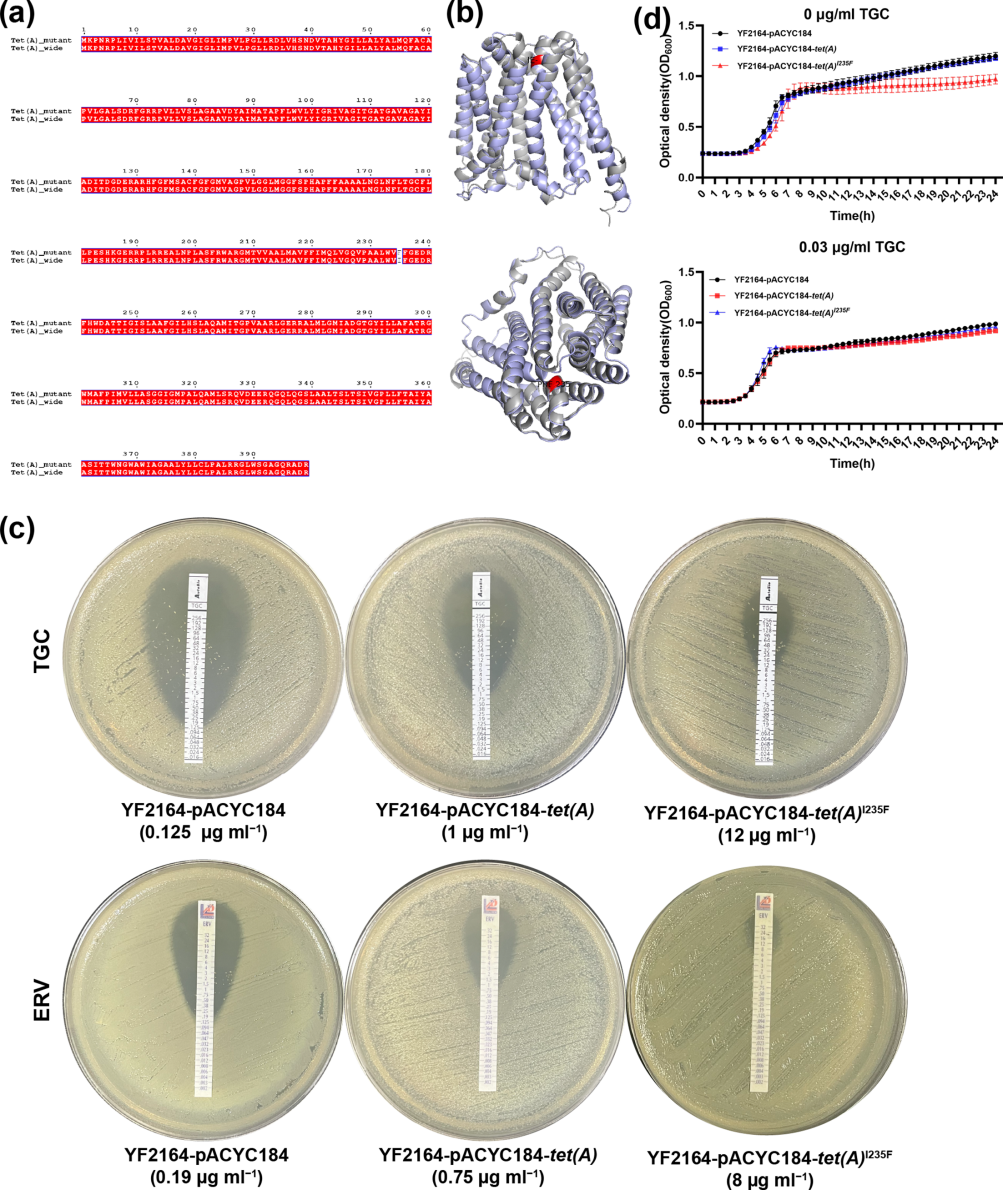
Tigecycline and eravacycline resistance phenotypes mediated by *tet*(A)^I235F^. (**a**) The amino acid sequence alignments of Tet(A) protein. (**b**) The three-dimensional structural model of Tet(A) protein (UniProt ID: P02981). The I235F mutation site was highlighted in red. (**c**) The E-test results of tigecycline (TGC) and eravacycline (ERV) for *K. pneumoniae* strains YF2164-pACYC184, YF2164-pACYC184-*tet*(A) and YF2164-pACYC184-*tet*(A)^I235F^. (**d**) Growth curves of *K. pneumoniae* strains YF2164-pACYC184, YF2164-pACYC184-*tet*(A) and YF2164-pACYC184-*tet*(A)^I235F^.

The AST results presented in Table 1 indicated that one isolate, YF2075, was resistant to tigecycline (MIC, 12 µg ml^−1^) and eravacycline (MIC, 6 µg ml^−1^). Furthermore, WGS analysis revealed that this isolate harboured a type 1 *tet*(A) gene with the I235F mutation (Fig. 3a, b). Previous research reported that CRKP strains evolve *tet*(A) mutations and develop high-level tigecycline resistance under tigecycline pressure [50,52]. To elucidate whether the *tet*(A) gene with the I235F mutation is associated with the tigecycline- and eravacycline-resistant phenotype of strain YF2075, a 1,750 bp amplicon containing the entire coding sequence of mutant *tet*(A)^I235F^ and the predicted upstream promoter was cloned into the pACYC184 vector (Fig. S2) and transformed into *K. pneumoniae* YF2164, which is sensitive to tigecycline and eravacycline with MICs of 0.25 and 0.19 µg ml^−1^, respectively. Meanwhile, the constructs harbouring pACYC184 and pACYC184-*tet*(A) [wild-type type 1 *tet*(A) gene cloned from the YF2190 strain] were incorporated for comparison. From the E-test results in Fig. 3(c), it is evident that 96- and 42-fold increases in the MICs of tigecycline and eravacycline, respectively, were achieved in the YF2164-pACYC184-*tet*(A)^I235F^ strain compared with the control strain YF2164-pACYC184, whereas YF2164-pACYC184-*tet*(A) strain exhibited sensitivity to tigecycline and eravacycline with MICs 1 and 0.75 µg ml^−1^, respectively (Fig. 3c). Besides, we observed a growth defect in the YF2164 strain harbouring the *tet*(A)^I235F^ mutation under drug-free conditions, and the growth defect was diminished under exposure to sub-MIC tigecycline (0.03 µg ml^−1^), indicating a potential fitness cost that may be ameliorated through adaptive compensation during drug exposure (Fig. 3d). Taken together, our findings showed that I235F was an essential mutation site for *tet*(A) to induce CRKP YF2075 isolate resistant to novel tetracycline antibiotics.

### The *tet*(A)^I235F^ mutation mediates reduced tigecycline treatment efficacy in a murine bloodstream infection model

**Fig. 4.**
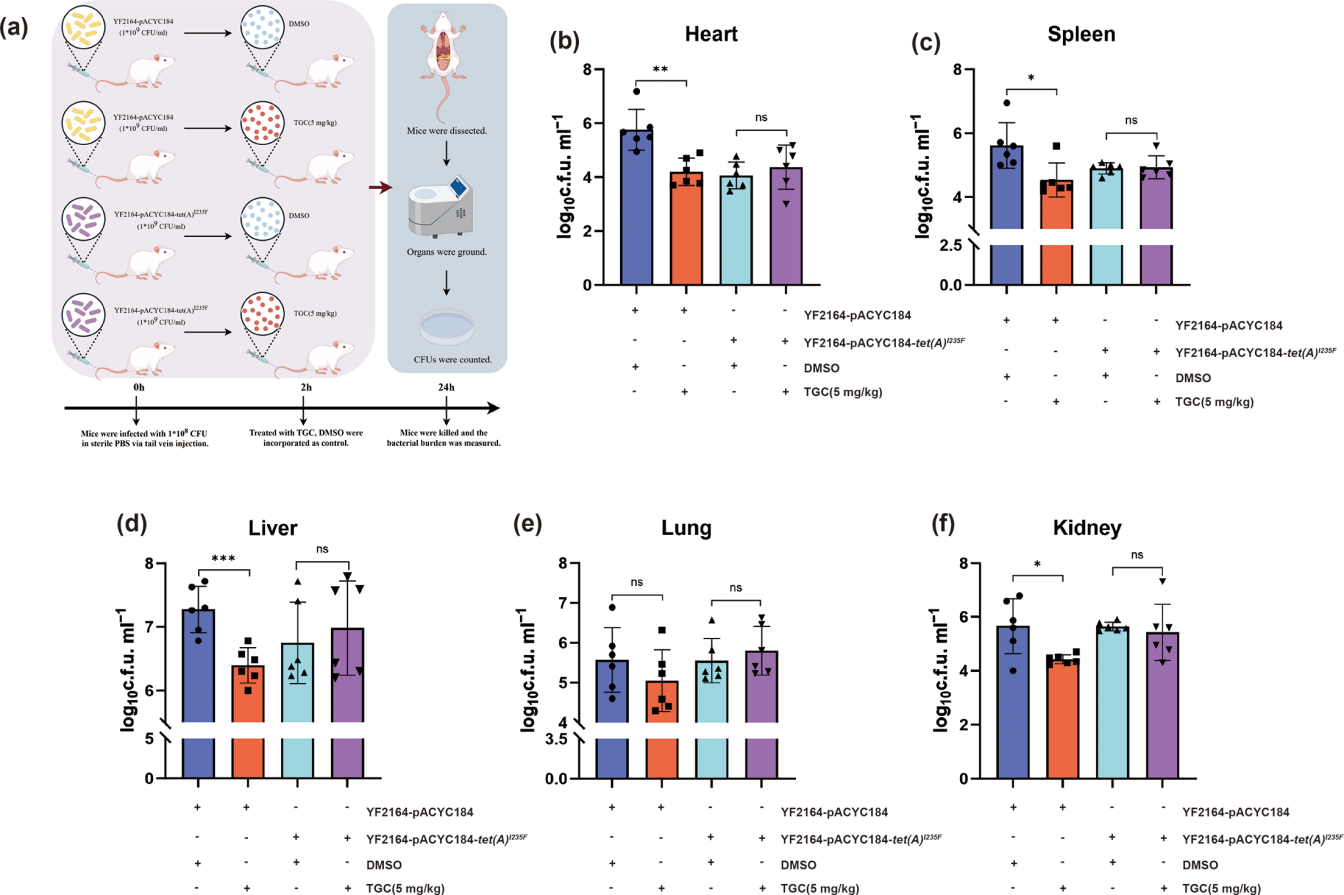
The *tet*(A)^I235F^ mutation compromises tigecycline efficacy *in vivo*. (**a**) Schematic of the experimental protocol for the murine bloodstream infection model treated with tigecycline (TGC, 5 mg kg^−1^). The groups treated with DMSO were incorporated as control. (**b–f**) Bacterial load in heart, spleen, liver, lung and kidney homogenates of respective groups.

The antimicrobial susceptibility results demonstrated that the *tet*(A)^I235F^ mutation conferred enhanced resistance to tigecycline and eravacycline. Therefore, we used a murine bloodstream infection model to further evaluate the *in vivo* treatment fitness cost of tigecycline in *K. pneumoniae* carrying the *tet*(A)^I235F^ mutation. *K. pneumoniae* strains were administered 1×10^8^ c.f.u. via the tail vein to healthy mice, and 5 mg kg^−1^ tigecycline was administered at 2 h post-infection to the treatment group (Fig. 4a). Mice were euthanized at 24 h, and visceral organs were aseptically excised and homogenized in sterile PBS (Fig. 4a). After tigecycline treatment, a significant reduction in bacterial viability was observed in the heart (reduce from 5.76±0.69 to 4.20±0.47 log_10_ c.f.u. ml^−1^, *P*<0.01) (Fig. 4b), spleen (reduce from 5.61±0.65 to 4.53±0.49 log_10_ c.f.u. ml^−1^, *P*<0.05) (Fig. 4c), liver (reduce from 7.27±0.34 to 6.39±0.25 log_10_ c.f.u. ml^−1^, *P*<0.001) (Fig. 4d) and kidney (reduce from 5.66±0.94 to 4.42±0.16 log_10_ c.f.u. ml^−1^, *P*<0.05) (Fig. 4f) for YF2164-pACYC184. Besides, a marginal reduction of 0.52±0.70 log_10_ c.f.u. ml^−1^ was detected in the lung (Fig. 4e). However, for mice infected with the YF2164-pACYC184-*tet*(A)^I235F^ strain, no significant reduction in bacterial burden was observed following tigecycline treatment (Fig. 4b–f), indicating the notable role of *tet*(A)^I235F^ in mediating diminished tigecycline efficiency to eliminate *K. pneumoniae in vivo*.

### Genomic characteristics of YF2075

**Fig. 5.**
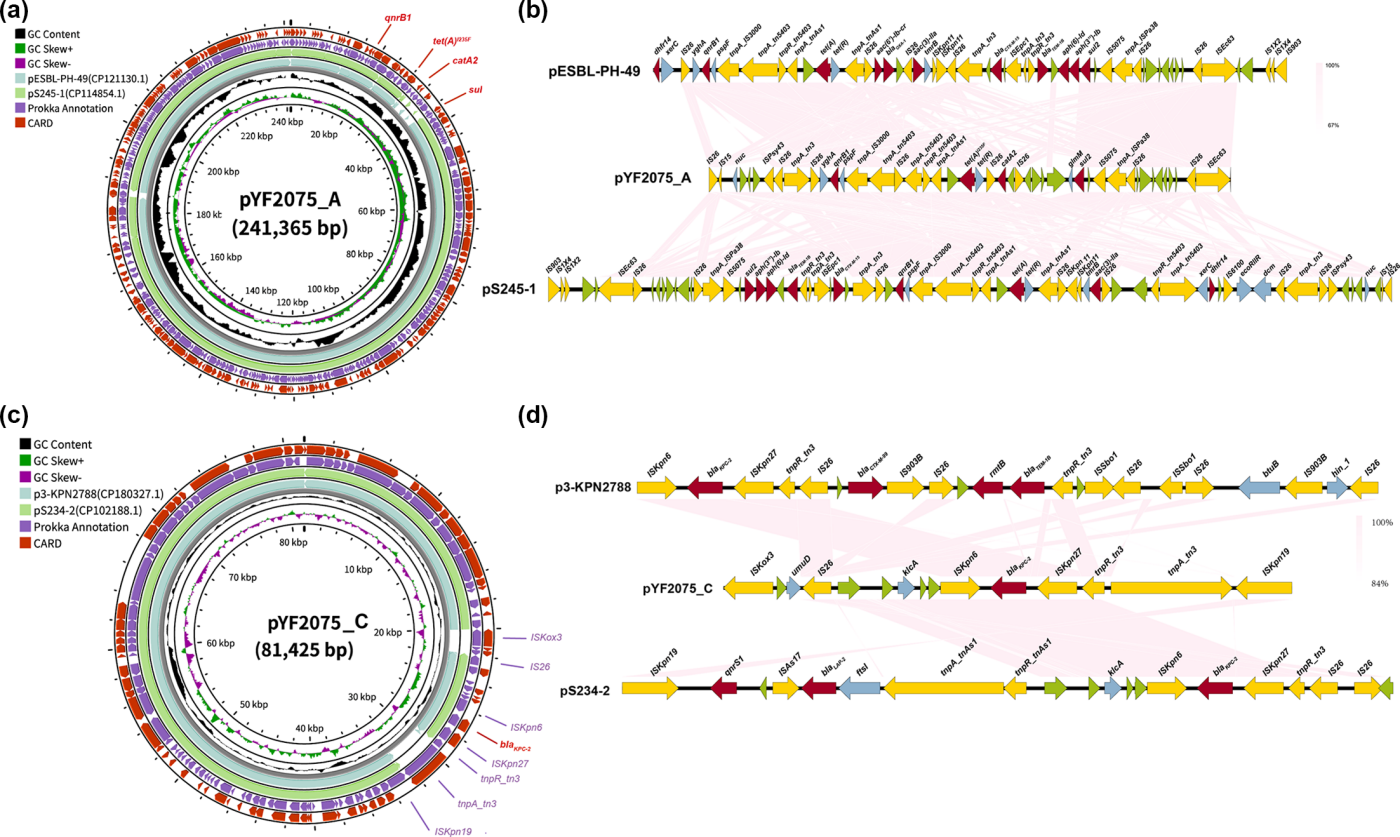
Comparative analysis of pYF2075_A and pYF2075_C plasmids with other reference plasmids. (**a**) Schematic map of plasmid pYF2075_A. (**b**) Alignment of the genetic environment surrounding *tet*(A)^I235F^ within pYF2075_A. The ARGs are shown in red, MGEs are shown in yellow, ORFs with specific functions are shown in blue and unidentified ORFs are shown in green. Pink shading indicates regions of shared homology among different elements. (**c**) Schematic map of plasmid pYF2075_C. (**d**) Alignment of the genetic environment surrounding *bla*_KPC-2_ within pYF2075_C.

YF2075 was selected for further sequencing using the PacBio platform. The genome size of YF2075 was 6,080,301 bp, comprising a 5,459,140 bp chromosome and five circular plasmids named as pYF2075_A to pYF2075_E (Table 3, Figs 5 and S4). pYF2075_A was an IncFII-type plasmid of 241,365 bp carrying resistance genes of *qnrB1*, *tet*(A)^I235F^, *catA2* and *sul2*. pYF2075_B was an IncFIB-type plasmid of 217,654 bp harbouring *msr(E*), *mph(E*), *aph(6)-Id*, *aph(3″)-Ib, qnrB4, bla*_DHA-1_*, sul1* and *armA. bla*_KPC-2_ harbouring plasmid pYF2075_C was an IncFII-type plasmid with a size of 81,525 bp. Besides, the resistance genes of *dfrA14* and *bla*_SHV-211_ were detected from pYF2075_D (type IncX3, 60,752 bp) and pYF2075_E (type CoIRNAI, 19,965 bp), respectively. Remarkably, a complete conjugation module (oriT, relaxase, T4CP and T4SS) was observed in plasmids pYF2075_A and pYF2075_C. As the above results demonstrate, the ability of pYF2075_C to transfer was verified by a conjugation assay, yielding 8.7×10^−3^ transconjugants per donor cell. However, we have not obtained transconjugants harbouring the pYF2075_A plasmid despite repeated conjugation assays. Sequence alignment showed that pYF2075_A shared 99.9% identity and 90% coverage with both pESBL-PH-49 (GenBank accession no. CP121130.1) and pS245-1 (GenBank accession no. CP114854.1) (Fig. 5a). Furthermore, the genetic environments of the *tet*(A) gene cluster revealed that these three identical plasmids shared a conserved backbone that simultaneously harboured other resistance genes (*qnrB1*, *catA2* and *sul2*) via mobile genetic elements, thereby mediating MDR phenotypes (Fig. 5b). pYF2075_C shared a similar plasmid backbone with p3-KPN2788 (GenBank accession no. CP180327.1) and pS234-2 (GenBank accession no. CP102188.1) (Fig. 5c). Moreover, the *bla*_KPC-2_ gene was located within the Tn6296 (truncated Tn6296) transposon, which comprises two insertion sequences, ISKpn27 and ISKpn6, a resolvase gene (tnpR_tn3) and the *bla*_KPC_ gene (Fig. 5d) [53].

**Table 3.** The plasmids in YF2075

	pYF2075_A	pYF2075_B	pYF2075_C	pYF2075_D	pYF2075_E
**Inc group**	IncFII	IncFIB	IncFII	IncX3	CoIRNAI
**Resistant genes**	*qnrB1, tet*(A)^I235F^*, catA2, sul2*	*msr(E), mph(E), aph(6)-Id, aph(3″)-Ib, qnrB4, bla*_DHA-1_ *, sul1, armA*	*bla*_KPC-2_	*dfrA14*	*bla*_SHV-211_
***oriT* (start…stop) (bp**)	195,728–195,777	–	52,147–52,228	–	–
**Relaxase (start…stop) (bp**)	225,184–230,442	37,911–40,859	52,584–54,512	44,311–45,471	18,538–19,269, 7,792–8,523
**T4CP (start…stop) (bp**)	222,872–225,184	40,849–42,975	14,195–16,387	31,269–33,104	16,574–18,538, 5,828–7,792
**T4SS (start…stop) (bp**)	195,170–231,247	22,390–42,975	182–16,387, 60,371–79,959	30,880–43,216	–
**Size (bp**)	241,365	217,654	81,525	60,752	19,965
**GC content (%**)	53	45	54	47	52
**Accession number**	PZ277059	PZ277060	PZ277061	PZ277062	PZ277063

oriT, the DNA at the origin of transfer; T4CP, type IV coupling protein.

### MgrB and PmrB mutations mediated colistin resistance in ST307 CRKP

**Fig. 6.**
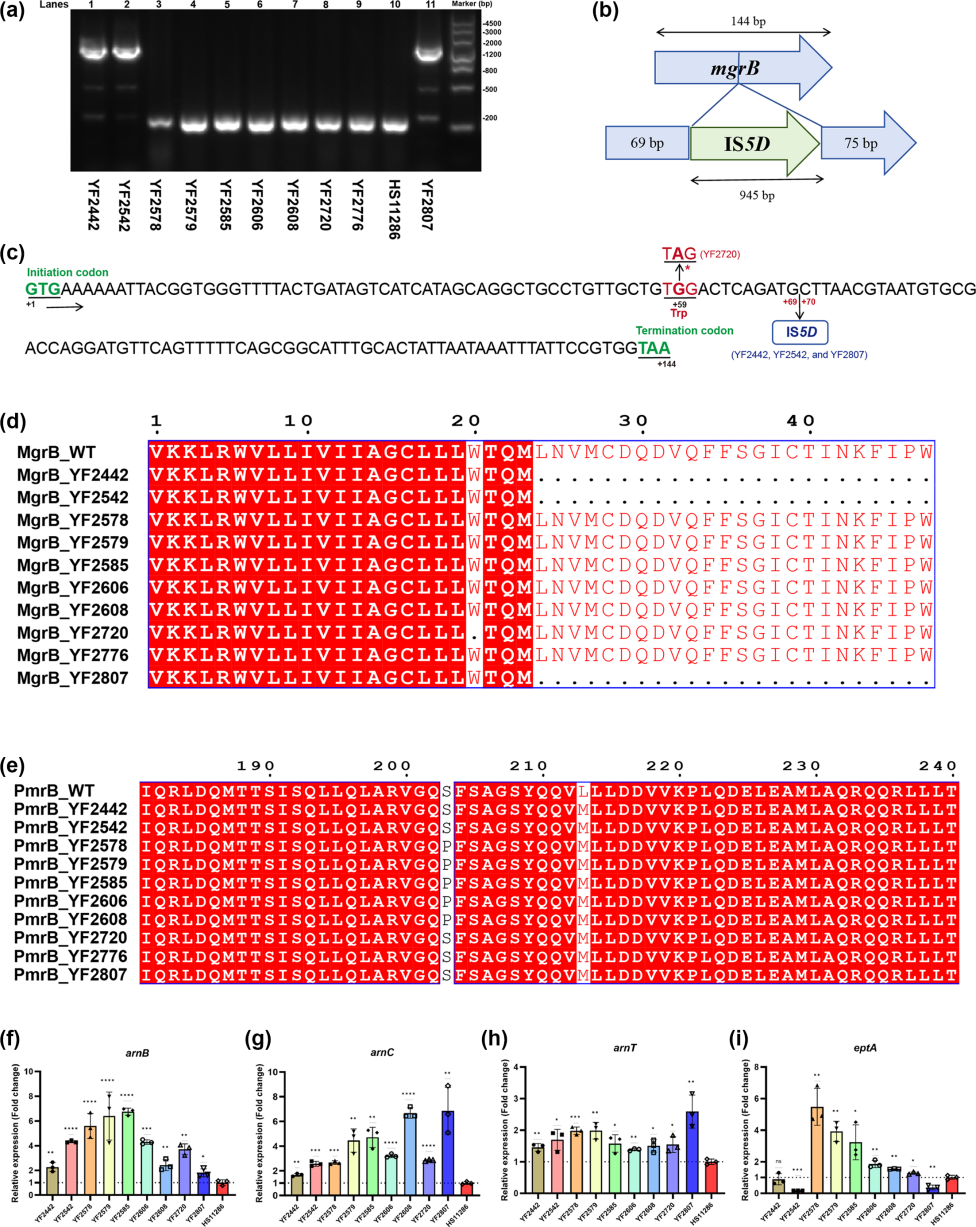
MgrB and PmrB mutations in colistin-resistant ST307 CRKP strains. (**a**) PCR of the *mgrB* gene in colistin-resistant isolates. The colistin-sensitive strain HS11286 (lane 10) was used as an amplification control. (**b**) *mgrB* gene structure of three isolates with IS*5D* insertion at position +69. (**c**) The nucleotide sequence of the *mgrB* gene. The insertion position of IS*5D* was highlighted in blue, and the mutation site of the *mgrB* gene in YF2720 was highlighted in red. (**d**) The amino acid sequence alignments of MgrB in relevant strains. (**e**) The amino acid sequence alignments of PmrB in relevant strains. (**f–i**) Relative expression levels of *arnB* (**f**), *arnC* (**g**), *arnT* (**h**) and *eptA* (**i**).

As mentioned above, ten isolates demonstrated resistance to colistin with MICs ranging from 4 to 32 μg ml^−1^. The molecular mechanism of colistin resistance was subsequently investigated. Given that all Col-R isolates were negative for *mcr* genes, we focused on examining chromosomal resistance mechanisms by sequencing key regulatory genes (Table 4). Among these ten isolates, the *mgrB* gene was disrupted in three isolates (YF2442, YF2542 and YF2807) as evidenced by the PCR product size (Fig. 6a). Identified by ISfinder analysis, we found that IS*5D* (IS*5* family) is inserted after 69 bp of the *mgrB* gene (Fig. 6b, c). Additionally, isolate YF2720 harboured the *mgrB* W20* mutation, resulting in the truncation of the MgrB protein (Fig. 6d). Furthermore, the *pmrB* S203P mutation, a functionally validated determinant of colistin resistance [54], was identified in 5 Col-R isolates (YF2578, YF2579, YF2585, YF2606 and YF2608) in our study (Fig. 6e). As shown in Fig. 6(e), the *pmrB* L213M mutation was identified in all strains; this mutation has been reported to be a silent mutation that does not disrupt PmrB protein function [55]. To assess the functional impact of these genetic alterations, we measured the expression of genes (*arnB*, *arnC*, *arnT* and *eptA*) involved in core lipid A modification by adding L-Ara4N and PEtN. Compared with colistin-sensitive *K. pneumoniae* strain HS11286 (MIC, 0.25 µg ml^−1^), all isolates showed increased expression of *arnB* (1.80- to 6.76-fold), *arnC* (1.67- to 6.86-fold) and *arnT* (1.39- to 2.59-fold) (Fig. 6f–h). Besides, isolates harbouring the *pmrB* S203P mutation showed diverse increased expression of *eptA* (1.55- to 5.48-fold) (Fig. 6i).

**Table 4.** Chromosomal mutations related to colistin resistance in Col-R ST307 CRKP isolates. WT, wide type

Strain	Mutation in polymyxin resistance-related genes				
	MgrB	PmrA	PmrB	PhoP	PhoQ
YF2442	IS*5D* insertion	WT	L213M	WT	WT
YF2542	IS*5D* insertion	WT	L213M	WT	WT
YF2578	WT	WT	S203P, L213M	WT	WT
YF2579	WT	WT	S203P, L213M	WT	WT
YF2585	WT	WT	S203P, L213M	WT	WT
YF2606	WT	WT	S203P, L213M	WT	WT
YF2608	WT	WT	S203P, L213M	WT	WT
YF2720	W20*	WT	L213M	WT	WT
YF2776	WT	WT	L213M	WT	WT
YF2807	IS*5D* insertion	WT	L213M	WT	WT

### Phylogenetic analysis of ST307 CRKP

**Fig. 7.**
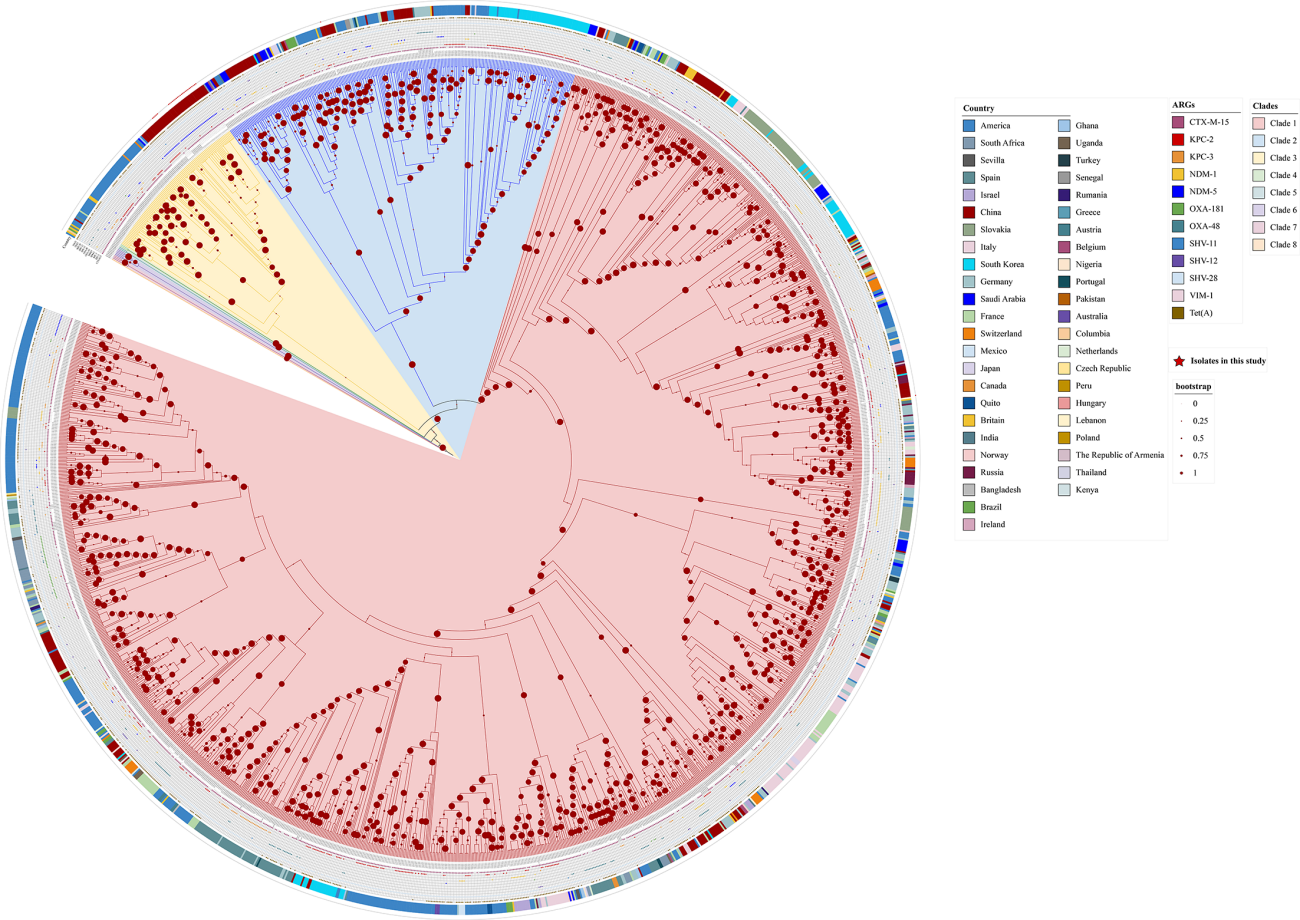
Phylogenetic analysis of global *K. pneumoniae* ST307 isolates. The genomes used for analysis include 1,701 international isolates from 46 countries (downloaded from the NCBI Pathogen Detection database) and 29 strains in this study. Clades were determined by clearly separating monophyletic branches with strong branch support, and isolates within the same clade shared high genomic relatedness. The solid circles on branches represent bootstrap values. Larger circles indicate higher statistical support for the corresponding node.

To determine the clonal relationships among these 29 ST307 CRKP strains, we mapped their genomes to the high-quality reference genome YF2075 and used paired distances to define clonal relatedness. The core genome SNP distance analysis showed that these 29 ST307 isolates differed by 14 to 2,409 core SNPs (Table S4) and were divided into 4 clusters (Fig. 1). Furthermore, we compared these isolates with 1,701 global ST307 *K. pneumoniae* strains. As shown in Fig. 7 1,730 ST307 isolates were divided into 8 clades. The isolates in this study, which were expected to be YF2279, were all grouped into clade 3, whereas YF2164 was classified in clade 1 (Fig. 7). Of the 1,730 ST307 isolates analysed, 22.77% (394/1,730) harboured *bla*_KPC-2_, whereas 6.99% (121/1,730) carried *bla*_NDM-5_ (Fig. 6). Besides, the prevalence rates of other *β*-lactamase genes specifically *bla*_CTX-M-15_, *bla*_KPC-3_, *bla*_NDM-1_, *bla*_OXA-181_, *bla*_OXA-48_, *bla*_SHV-11_, *bla*_SHV-12_, *bla*_SHV-28_ and *bla*_VIM-1_ were 83.12% (1,438/1,730), 13.00% (225/1,730), 9.19% (159/1,730), 2.95% (51/1,730), 8.44% (146/1,730), 0.35% (6/1,730), 1.73% (30/1,730), 96.46% (1,686/1,730) and 0.46% (8/1,730), respectively (Fig. 7). These results suggest that *K. pneumoniae* ST307 is emerging as a significant high-risk antimicrobial-resistant clone worldwide. Furthermore, we found that the prevalence of *tet*(A)-positive ST307 *K. pneumoniae* strains was notably high, reaching 58.44% (1,011/1,730) (Fig. 7). This suggests an increased risk of *tet*(A) site mutations that may confer resistance to novel tetracycline antibiotics in this clone.

**Fig. 8.**
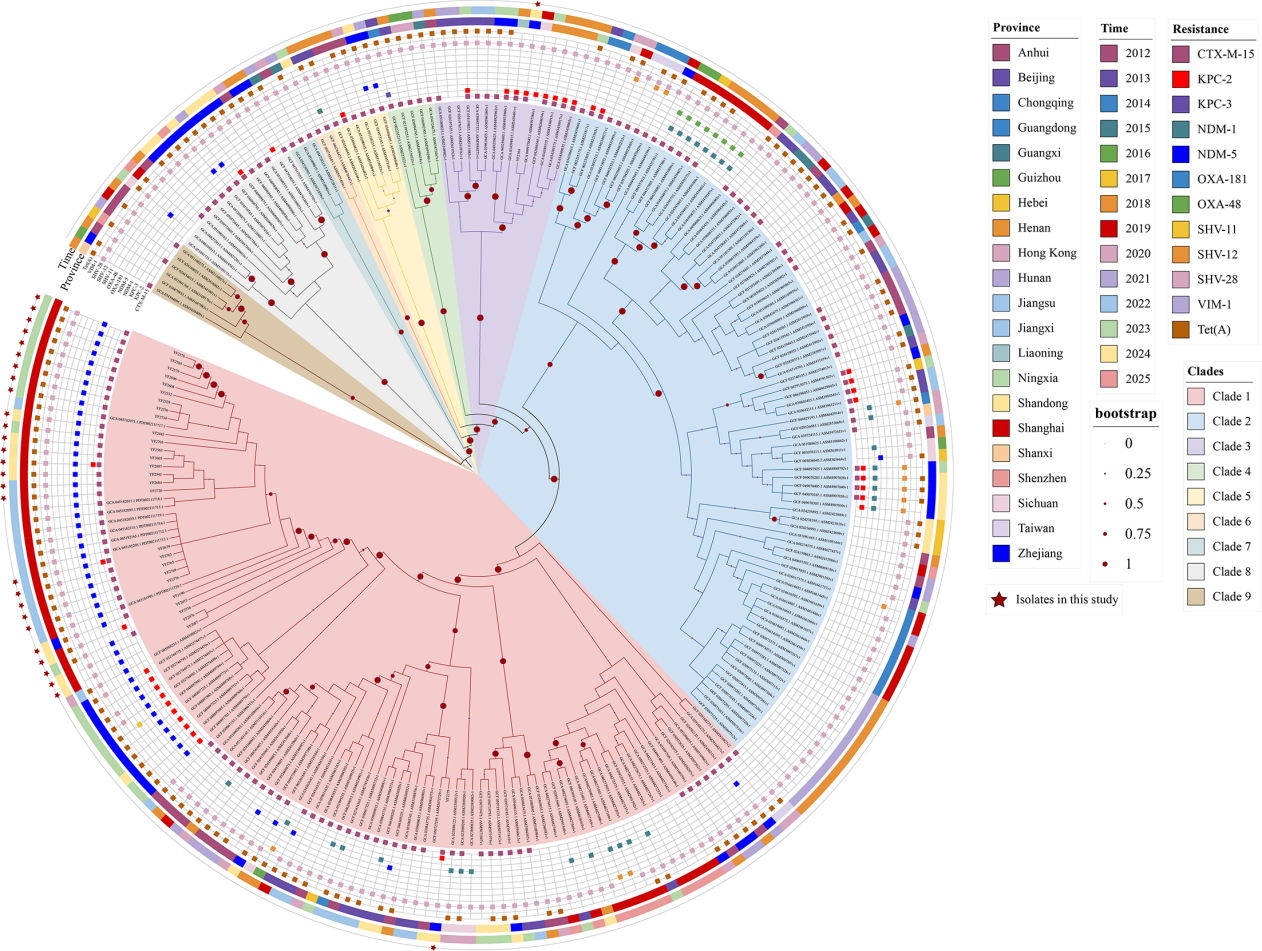
Phylogenetic analysis of Chinese *K. pneumoniae* ST307 isolates. A total of 213 *K*. *pneumoniae* ST307 strains isolated from 21 provinces in China and 29 strains in this study were incorporated for analysis. Clades were defined based on well-separated monophyletic branches. These clades represent a distinct, independent classification rather than subclades derived from the eight major clades shown in Fig. 7. The solid circles on branches represent bootstrap values. Larger circles indicate higher statistical support for the corresponding node.

Fig. 7 shows that our 29 ST307 strains were phylogenetically close to strains from China. Thus, we mapped the phylogenetic tree of 29 isolates from this study and 213 strains from 21 provinces in China. As shown in Fig. 8, 27 strains isolated from hospitals A and B (both located in Shanghai) were phylogenetically close to the *bla*_NDM-5_-carrying clone that was outbroken in Shanghai [19], suggesting clonal transmission among 3 different hospitals. YF2164, the strain carrying *bla*_KPC-2_, exhibited a close phylogenetic affinity with another five KPC-2-producing CRKP ST307 strains isolated from Sichuan and Henan (Fig. 8). In addition, YF2279 clustered phylogenetically with previously sequenced non-CRKP strains from Shanxi Province (Fig. 8). Among the 242 analysed ST307 strains, 15.29% (37/242) carried *bla*_KPC-2_ and 0.41% (1/242) carried *bla*_KPC-3_, suggesting that the prevalence of KPC-producing ST307 strains was considerably lower in China than the global level (Fig. 8). However, our analysis revealed a markedly higher prevalence of *bla*_NDM-1_ (12.81%, 31/242) and *bla*_NDM-5_ (23.14%, 56/242) among Chinese ST307 isolates compared with global surveillance data (Fig. 8). In terms of collection time, 65.79% (25/38) *bla*_KPC_-carrying ST307 strains were detected after 2023, and 54.02% (47/87) NDM-producing ST307 isolates were collected between 2023 and 2025 (Fig. 8). These findings alert us that NDM-producing ST307 was emerging as a high-risk, XDR clone in China following the coronavirus disease 2019 (COVID-19) pandemic.

### The pathogenicity and virulence determinants of ST307 CRKP

**Fig. 9.**
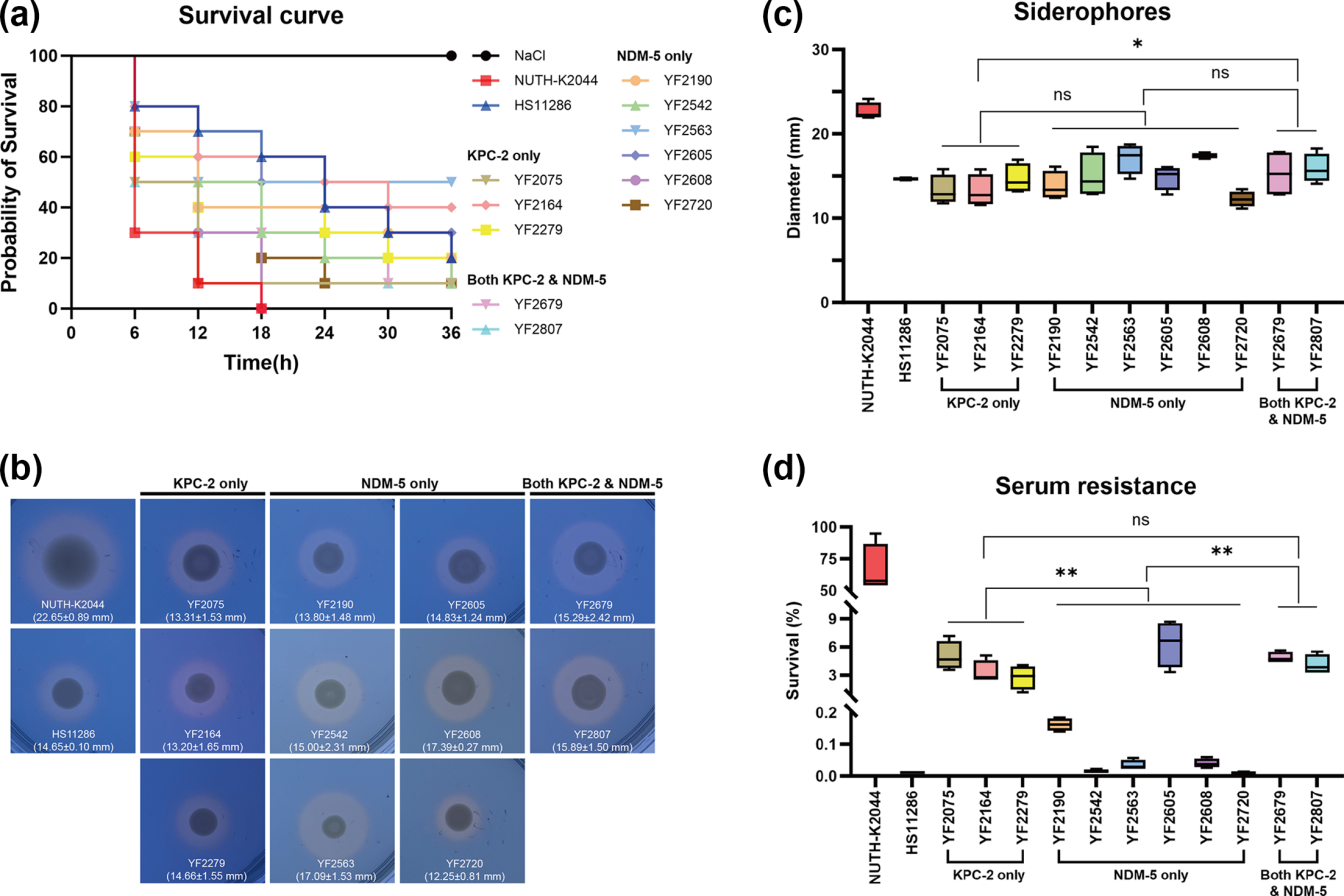
The virulence phenotype of *K. pneumoniae* ST307 isolates. (**a**) Survival curves of *G. mellonella* infected with *K. pneumoniae* ST307 isolates, HS11286 and NUTH-K2044. NaCl, normal saline. (**b, c**) Siderophores production determined by CAS agar plate. (**d**) Survival rate (%) of strains in serum resistance assay.

As shown in Fig. 1, all ST307 CRKP isolates in our study were found to harbour the virulence-associated genes involved in siderophore synthesis and transport (*entA*, *entB*, *entE*, *entS*, *fepA*, *fepB*, *fepC*, *fepD* and *fepG*), pilus/adhesin formation (*yagZ/ecpA*, *yagY/ecpB*, *yagX/ecpC*, *yagW/ecpD*, *yagV/ecpE*, *ykgK/ecpR*, *fimA*, *fimE*, *clfA* and *clfB*), type II secretion system (*xcpA/pilD* and *xcpR*), outer membrane protein expression (*ompA*) and magnesium iron transport (*mgtB* and *mgtC*). Virulence scores for the 29 ST307 CRKP were determined using Kleborate v3.2.4. Most strains exhibited virulence scores of 0 (25/19, 86.2%) (Table S5). Only three isolates carried a virulence score of 1 (YF2679, YF2279 and YF2164), and just one strain (YF2807) had a virulence score of 4 (Table S5). These results indicate that most ST307 CRKP isolates in this research possessed low virulence potential. To further analyse the virulence characteristics of these isolates, we randomly selected six *bla*_NDM-5_-positive ST307 CRKP strains and compared them with three *bla*_KPC-2_-carrying isolates, two ST307 CRKP producing both NDM-5 and KPC-2, NUTH-K2044 (ST23 hypervirulent *K. pneumoniae* strain) and HS11286 (*bla*_KPC-2_-positive ST11 *K. pneumoniae* strain). Firstly, *G. mellonella* infection model was applied to evaluate pathogenicity *in vivo*. At 36 h post-infection, YF2075 (10%), YF2542 (10%), YF2608 (0%), YF2679 (10%), YF2720 (10%) and YF2807 (10%) showed stronger virulence than HS11286 (negative control, 20%), while the pathogenicity of YF2164 (40%), YF2563 (50%) and YF2605 (30%) were slightly lower than HS11286 (Fig. 9a). However, ST307 CRKP strains all demonstrated lower virulence than NUTH-K2044, which resulted in a 0% survival rate after 18 h post-infection (Fig. 9a). Quantitative siderophore assays indicated that ST307 CRKP produced comparable siderophores with HS11286 (Fig. 9b), but we observed that the siderophore synthesis capacity of the group producing both NDM-5 and KPC-2 was slightly stronger than that of the isolates only carrying *bla*_KPC-2_ (Fig. 9c). What is more, we performed serum resistance assays to evaluate the capability of ST307 CRKP isolates to resist the lethal effect of host immune molecules. Notably, except YF2542 (0.015±0.005%) and YF2720 (0.008±0.003%), all tested ST307 CRKP strains demonstrated higher levels of serum resistance than HS11286 (0.011±0.004%) (Fig. 9d). Besides, we found that the isolates carrying *bla*_KPC-2_ only (3.68±1.51%) or both harbouring *bla*_KPC-2_ and *bla*_NDM-5_ (4.48±0.83%) exhibited significantly stronger serum resistance capacity than those only carrying *bla*_NDM-5_ (1.10±2.50%) (Fig. 9d). As demonstrated by the serum resistance assay results of four consecutive isolates from a single patient (YF2563, YF2542, YF2605 and YF2807), ST307 CRKP strains could improve their tolerance to the immune system during the course of *in vivo* evolution (Fig. 9d). The survival rates of earlier collected strains YF2563 and YF2542 were 0.03±0.01% and 0.02±0.01%, respectively, while the subsequently extracted isolates YF2605 and YF2807 exhibited survival rates above 4% (Fig. 9d). Taken together, these results suggested that the ST307 CRKP isolates in our research exhibited relatively low virulence, whereas isolates with distinct resistance profiles displayed different patterns of tolerance to immune killing by the host.

## Discussion

In the USA and European countries, *K. pneumoniae* ST258 is the predominant ST responsible for the dissemination of carbapenem resistance, and ST11 has contributed significantly to the spread of carbapenemase genes in China [56]. However, various studies reported that several new lineages exhibiting XDR phenotypes have emerged to achieve global dissemination [8,9]. In this study, we reported that ST307, a super-clone replacing ST258 as the most prevalent clone associated with multidrug resistance genes in some European countries [11,12], exhibited the ability to drive nosocomial CRKP outbreaks in China. In contrast to the widespread dissemination of ST307 CRKP in other countries, the ST307 CRKP strains carrying carbapenemase genes were sporadically reported in China. Our analysis of public genomic data revealed that *K. pneumoniae* ST307 is always associated with several AMR determinants, such as CTX-M-15, KPC, NDM and OXA-48, leading to worldwide nosocomial CRKP outbreaks. Furthermore, we observed a significant increase in the prevalence of ST307 CRKP in China following the COVID-19 pandemic, urging the need to control the spread of this high-risk clone.

From the phylogenetic analysis, we found that 27 ST307 CRKP isolates in this study were phylogenetically close to the *bla*_NDM-5_-carrying isolate outbreak in Shanghai in 2022 [19]. In our study, the first ST307 CRKP strain (YF2763) was isolated on 29 August 2022, whereas the initial detection of ST307 CRKP in their research was on 5 September 2022, representing an extremely close temporal interval between the two independent findings. A notable similarity between the two studies was the ward-specific outbreak of ST307 CRKP, which occurred in an identical clinical unit in both cases. In hospital A, our investigation documented a prolonged presence of ST307 CRKP in the post-operative monitoring ward, with isolates consistently recovered over 18 months from August 2022 to April 2024. Notably, our research further reveals that ST307 CRKP strains responsible for this outbreak can acquire the *bla*_NDM-5_ or *bla*_KPC-2_ gene via plasmid-mediated horizontal transfer and even obtain both *bla*_NDM-5_ and *bla*_KPC-2_ genes simultaneously during the course of evolutionary adaptation. We collected four consecutive ST307 CRKP strains (YF2563, YF2542, YF2605 and YF2087) from a lung transplant recipient. The first isolated strain, YF2563, carries *bla*_NDM-5_ but is sensitive to colistin. However, with the administration of colistin, the subsequent isolates recovered from this patient evolved into colistin-resistant strains and eventually developed into a variant that harbours both *bla*_NDM-5_ and *bla*_KPC-2_ genes. In China, co-producing KPC-2 and NDM CRKP isolates (KPC-2-NDM-CRKPs) have been rarely identified with different STs (ST11, ST15, ST86 and ST464) [57,62]. In this study, we reported that ST307 *K. pneumoniae* possesses the inherent capacity to evolve into KPC-2-NDM-CRKPs and then poses a significant threat to public health.

When international ST307 isolates were included in phylogenetic relatedness analysis, 27 isolates clustered with the lineage isolated from the USA, indicating the potent capacity of ST307 CRKP to drive transnational outbreaks. Through KBseq high-throughput sequencing (Sangon Biotech), we found that in the genome of YF2087, *bla*_NDM-5_ was located on a 45,181 bp IncX3 plasmid, whereas in the genome of YF2075, *bla*_KPC-2_ was carried by an 81,425 bp IncFII plasmid. It has been reported that *bla*_NDM_ is often located on the IncX3 plasmid, which serves as the primary vehicle for *bla*_NDM_ transmission [63]. Given that the IncX3 plasmid exhibited low fitness cost, outstanding conjugation ability and high stability, it facilitates the rapid dissemination of *bla*_NDM-5_ in China [49]. A previous study demonstrated that the IncFII KPC-2 plasmid exhibited higher expression of transfer-associated genes, leading to a higher conjugation frequency [64]. It is worth noting that the IncFII KPC-2 plasmid from YF2075 displayed practical conjugation ability. Therefore, it is crucial to monitor the IncFII plasmid-mediated dissemination of the *bla*_KPC-2_ gene in this high-risk clone.

Tigecycline, eravacycline and colistin are among the few antimicrobials remaining active against CRKP. However, ST307 CRKP is emerging as a high-risk clone that not only is resistant to carbapenems but also has developed resistance to novel antimicrobials through genetic mutations. The Tet(A) protein, a major facilitator superfamily efflux pump, is critical for tigecycline resistance: mutations in *tet*(A) may result in increased intracellular tigecycline accumulation, thereby contributing to resistance development [65]. Researchers found that *tet*(A) mutations predominantly accumulated in transmembrane domains of the Tet(A) efflux pumps, most likely leading to protein structure changes and decreasing the efficiency of transporting tetracycline antibiotics [65]. Linkevicius *et al*. have confirmed that *tet*(A) mutants of I235F show increased tigecycline MIC compared to the control [65]. In this study, we further confirmed that the plasmid-borne *tet*(A)^I235F^ gene is a major determinant of eravacycline resistance in ST307 CRKP. In strain YF2075, *tet*(A)^I235F^ was located on a 241,365 bp IncFII plasmid carrying a complete conjugation module. Comparative analysis showed that this plasmid shares structural similarity with two other *tet*(A)-bearing plasmids, all of which harbour multiple antibiotic resistance genes. This suggests that *tet*(A)-bearing plasmids confer a higher risk of simultaneous resistance to tigecycline and carbapenems. Notably, *K. pneumoniae* carrying *tet*(A) is more prone to developing tigecycline resistance under selective pressure [50]. Phylogenetic analysis revealed the widespread distribution of *tet*(A) among global ST307 *K. pneumoniae* isolates. Thus, vigilance is warranted against strains harbouring evolved *tet*(A) mutations, as these will exacerbate the therapeutic dilemma. It is well established that antibiotic resistance is often associated with a fitness cost, typically observed as a reduced bacterial growth rate [66]. A previous study demonstrated that type 1 *tet*(A) significantly impairs the growth of clinical *K. pneumoniae*, imposing a substantial biological fitness cost under antibiotic-free conditions both *in vitro* and *in vivo* [51]. In this study, we found that the *tet*(A) I235F mutation can cause a measurable growth defect in CRKP. This phenotype may reflect impaired proton coupling efficiency and inner membrane integrity, thereby dissipating the proton-motive force essential for core cellular processes and triggering envelope stress responses that redirect resources away from growth [67]. However, further studies are required to verify this mechanism.

Recent studies have shown that mutations in PmrAB and PhoPQ as well as disruptions of the negative regulator MgrB are key contributors to colistin resistance in *K. pneumoniae* [68,69]. However, the determinants of colistin resistance vary among strains with different lineage-specific backgrounds [54]. Here, we found that ST307 CRKP are prone to colistin resistance mediated by *mgrB* and *pmrB* mutations. Although a previous study has reported IS*5D* insertion disrupting the *mgrB* gene in carbapenem-resistant *Enterobacter cloacae* complex strains [70], our study first identified this disruption pattern in CRKP. Additionally, this is the first report of the *mgrB* W20* mutation, underscoring the importance of WGS for surveillance of novel mutation sites. The expression of the *arn* operon and *eptA* is directly activated by the PmrAB two-component system [32]. Nevertheless, strains carrying the identical *pmrB* S203P mutation exhibited distinct expression levels of these downstream genes. This discrepancy arises from diverse genetic backgrounds among isolates, including intrinsic genetic variations, accessory genes and distinct regulatory network [71].

In conclusion, our study investigated the evolution of ST307 CRKP by using a combination of WGS and epidemiologic techniques. The epidemiological investigation showed that ST307 is a high-risk clone responsible for the nosocomial spread of CRKP. We further elucidated the dissemination of ST307 CRKP in China, highlighting the urgent need for enhanced surveillance of its outbreak, even in regions previously presumed to have low prevalence of this clone. Furthermore, we identified a crucial mutation site in *tet*(A) gene that confers resistance to both tigecycline and eravacycline in *K. pneumoniae* and elucidated the molecular mechanism of colistin resistance in ST307 CRKP. These findings underscore the necessity for stringent antibiotic stewardship.

## Abbreviations

AMR: antimicrobial resistance
ARGs: antimicrobial resistance genes
AST: antimicrobial susceptibility test
CAS: chrome azurol S
CLSI: Clinical and Laboratory Standards Institute
COVID-19: coronavirus disease 2019
CRKP: carbapenem-resistant *Klebsiella pneumoniae*
ESBL: extended-spectrum *β*-lactamase
KPC: *Klebsiella pneumoniae* carbapenemase
LB: Luria–Bertani
LPS: lipopolysaccharide
MDR: multidrug-resistant
MGEs: mobile genetic elements
MIC: minimum inhibitory concentration
MLST: multilocus sequence type
ST: sequence type
WGS: whole-genome sequencing
XDR: extensively drug-resistant
